# Enhanced production and structural characterization of exopolysaccharide from *Sporocarcina psychrophile* MTCC 2908 through two step optimization for therapeutic evaluation

**DOI:** 10.1038/s41598-025-10392-5

**Published:** 2025-07-17

**Authors:** Baidurja Biswas, Subbalaxmi Selvaraj, Kannan Natarajan

**Affiliations:** Department of Biotechnology, Manipal Institute of Technology (MIT), Manipal Academy of Higher Education (MAHE), Manipal, 576104 India

**Keywords:** Exopolysaccharide, *Sporocarcina psychrophila* MTCC–2908, Statistical optimization, Characterization, Antioxidant, Reducing power potential, Biotechnology, Industrial microbiology, Industrial microbiology

## Abstract

The production and extraction of Exopolysaccharide (EPS) from the bacterial strain *Sporocarcina psychrophila* (MTCC–2908) was carried out in submerged fermentation conditions, yielding 1.22 g/L in 48 h at unoptimized conditions. To enhance EPS yield, a two-stage optimization strategy was employed using Plackett-Burman design (PBD) for parameter screening, followed by central composite design (CCD) for optimization. To further enhance the yield of EPS. Ten fermentation parameters were initially screened via PB design, of which five (Glucose, NH_4_Cl, K_2_HPO_4_, MgSO_4_ 7H_2_O, MgSO_4_·H_2_O) were identified as significant. These were further optimized using CCD. Under these conditions, maximum EPS production reached 21.62 g/L, representing a 17-fold increase compared to unoptimized conditions. Further, Lyophilized EPS was characterized using techniques Fourier Transform Infrared, Thermogravimetric Analysis, Atomic Force Microscopy, Scanning Electron Microscope, X-ray diffraction, total antioxidant capacity, and the reducing activity of the EPS. The purified EPS showed a moderate amount of antioxidant capacity and reducing activity, which may have a potent application in the pharmaceutical industry. The result of this study could serve as a promising candidate for further development of therapeutic applications of the EPS produced from *Sporocarcina psychrophila* MTCC–2908.

**Supplementary Information:**

The online version contains supplementary material available at 10.1038/s41598-025-10392-5.

## Introduction

Exopolysaccharides (EPS) are long chains of sugar units such as glucose, galactose, and rhamnose found in various bacteria^1^. Polysaccharides may be embedded in the cells of diverse bacterial species including storage polysaccharides for example glycogen^2^. Structural polysaccharides that comprise cell walls as well as exocellular polysaccharides leading to the formation of glycocalyces. These exocellular polysaccharides include capsular polysaccharides and EPS^2^. Some of these EPS have been shown to possess valuable properties such as biodegradability, non-toxicity, biocompatibility, cell adhesion, environmental protection, and energy storage^3^. Heteropolysaccharides like hyaluronic acid and homopolysaccharides such as dextran are examples of EPS that exhibit monosaccharide composition-based classification^4^. This is because their unique rheological properties make them highly useful in industries such as food, cosmetics, pharmaceuticals and textiles^4^. Other applications include using these polymers as emulsifiers and stabilizers among other functions such as moisture retention^4^. EPS has potential to wound healing, environmental applications such as heavy metal tolerance and metal biosorption^5,6^.

Simple sugars such as glucose and sucrose commonly serve as the primary carbon and energy sources, whereas amino acids or ammonium salts serve as nitrogen sources. They are required for microbial growth and production of biomolecules^7^. The ratio of carbon and nitrogen (C:N) available in environmental conditions has an important role in EPS production; low nitrogen levels enhance EPS production^8^. There are predominant routes to the EPS biosynthesis in bacterial species, the Wzx/Wzy-dependent, ABC transporters-dependent, synthase-dependent, and extracellular synthesis mediated by sucrase protein^9,10^.

EPS in nature can be produced through different fermentation techniques such as Solid state fermentation (SSF) and submerged fermentation (SmF), which are utilized to produce exopolysaccharides and other beneficial substances under controlled environments. The SmF was selected over SSF due to its superior control over critical process parameters such as pH, temperature, aeration, and agitation, which are essential for consistent and enhanced EPS production^1,4^. SmF also facilitates easier downstream processing, uniform nutrient distribution, and scalability, making it more suitable for industrial applications and large-scale bioprocess optimization^8^^9^. Fermentation leverages the metabolic pathways of microorganisms to convert complex substrates into value-added secondary metabolites, such as exopolysaccharides (EPS). In microbial systems, the type and yield of bioactive metabolites are species-specific and significantly influenced by environmental parameters such as nutrient availability, pH, temperature, and oxygen levels. These conditions modulate the biosynthesis of industrially valuable compounds, including antibiotics, pigment enzymes, and antioxidants^11^. Some of the bacterial strains that secrete EPS were reported, such as *Lactobacillus plantarum*^1^ *Bacillus cereus*^12^ *Lacticaseibacillus paracasei* CIDCA 8339, CIDCA 83,123 and CIDCA 83,124^13^, *Leuconostoc pseudomesenteroids* 56 and *Weissella cibaria* 21 and 64^14^, and *Alkalibacillus* sp.w^15^ *Bacillus haynesi* CamB6^16^, and *Streptococcus thermophilus*^17^.

EPS has different functionalities like antioxidant activity, antitumor effects, immunomodulatory properties, and acid hydrolysis properties. Several parameters affect the antioxidant potential of bacterial exopolysaccharides (EPS), such as fermentation conditions, the bacterial growth and fermentation kinetics, the type of monosaccharides, glycosidic linkage patterns, degree of branching^18,19^. Acid hydrolysis is commonly employed to analyse the monosaccharide composition of EPS by cleaving glycosidic linkages to release individual sugar units. Variations in monosaccharide compositions contribute to functional differences among EPS types. For instance, glucose-rich EPS have demonstrated strong scavenging activity against superoxide and DPPH radicals^19^. Similar to their function in modulating monosaccharide proportion in structure heteropolysaccharide, bacterial fermentation also plays a significant role in determining the structure and corresponding radical DPPH scavenging/Hydroxyl radical scavenging for EPS, such as having a high mannose to galactose ratio has potent hydroxyl radical scavenging and DPPH reduction abilities^20^. The antioxidant properties of EPS, composed of glucose, galactose, and rhamnose as the main monosaccharide constituents, have also been reported. Bacterial fermentation conditions, such as pH, govern EPS structure and hence affect antioxidant properties. This is made possible through regulation of the proportion of monosaccharides in the EPS that occurs as a result of varying pH leading to differences in the level of antioxidant activity exhibited by such polysaccharides^21^. These parameters indicate that different bacterial EPS could be used for various applications having antioxidant ability as indicated^22^.

The ability of exopolysaccharides (EPS) to fight against microbes is influenced by their features. EPS consists of groups, like carbonyl phosphate and hydroxyl groups that interact with bacterial cell membranes to exhibit antimicrobial effects^23^. Research indicates that EPS made up of glucose and rhamnose shows properties against pathogens such as *Staphylococcus aureus*, *Bacillus subtilis*, and *Bacillus pertussis*^24^. Furthermore, EPS produced by bacteria with glucose mannose, galactose, fucose, and uronic acid fractions have displayed effects against various pathogens. The composition of EPS including the types of monosaccharides and the presence of groups plays a crucial role in determining their antimicrobial activity which make them a promising options for antimicrobial applications^22^.

Exopolysaccharides (EPS) from a variety of bacterial species have been the subject of much investigation, but little is known about novel or underreported microbial strains that can produce EPS with strong reducing and antioxidant capabilities. Furthermore, psychrotolerant or lesser-known genera like *Sporocarcina* are substantially ignored in the majority of the research currently in publication, which concentrates on well-studied genera like *Lactobacillus*^1^ *Streptococcus*^16^ and *Bacillus*^12^. Additionally, there aren’t many thorough investigations that include purification, structural characterization, optimum fermentation conditions, and assessment of bioactivities including antioxidant capacity in a single workflow for EPS made from these novel strains. Despite growing interest in mesophilic bacteria as novel EPS producers, *Sporosarcina psychrophila*, a psychrotolerant, Gram-positive, spore-forming bacterium, remains underexplored. Originally isolated from cold environments, *S. psychrophila* is known for its unique enzymatic and metabolic adaptations, which may facilitate EPS biosynthesis under low-temperature or nutrient-stress conditions. For cryoprotection, food preservation, and cosmetics, its psychrotolerance facilitates the creation of EPS with improved solubility, stability, and function at low temperatures. Rare sugars or new connections may be present in these EPS, providing special structural, rheological, or bioactive qualities that are advantageous in cold-chain applications. While *Sporosarcina* species have been previously noted for urease activity and bioremediation potential^25^ there is a lack of comprehensive studies on their capacity for EPS production. To date, there is no detailed report evaluating the EPS yield, structural properties, or antioxidant activity of EPS from *S. psychrophila*. This gap presents an opportunity to explore novel EPS with therapeutic potential from psychrotolerant sources.

In this work, the submerged fermentation process for producing EPS from *Sporosarcina psychrophila* MTCC-2908 was optimized. To assess the pharmacological potential of the crude EPS, it was purified, characterized, and its antioxidant and reducing activities were examined. To the best of our knowledge, this is the first research showing that *S. psychrophila* MTCC-2908 produces EPS with bioactive qualities, which adds to our understanding of the possible uses of psychrotolerant bacterial EPS.

## Materials and methods

### Materials

All chemicals, reagents, and media components used in this study were of analytical grade and procured from Loba Chemie, Sigma-Aldrich, and HiMedia,

### Bacterial strain and preservation

The bacterial strain *Sporocarcina psychrophila* MTCC-2908 was procured from the cell culture facility of the Institute of Microbial Technology (IMTECH), Chandigarh-India, in a lyophilized form. The Glycerol stock of the strain was prepared and preserved at − 80 °C for further use.

### Preparation of inoculum

**Fig. 1:**
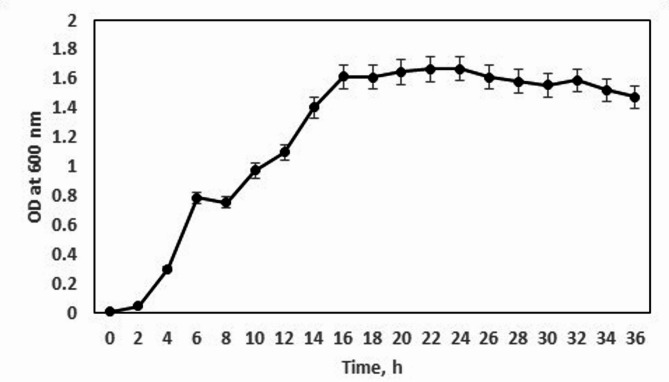
Growth curve of *Sporocarcina psychrophila* MTCC-2908 in nutrient broth.

The bacterial strain was inoculated into nutrient broth (NB) media and retrieved the active nature of the culture. The strain was further sub-cultured into 100 mL NB and stored at 4 °C for maintenance. During the course of inoculum preparations, the growth profile of the *Sporocarcina psychrophila* MTCC-2908 was constructed as shown in Fig. 1. The mid-exponential culture of 10 h was used for inoculation.

### Submerged fermentation for EPS production

A 100 mL fermentation media consisting of (% w/v): Glucose, 20; NH_4_Cl, 6; K_2_HPO_4_, 1.1; MgSO_4_·7H_2_O, 0.3; MgSO_4_·H_2_O, 0.3; CaCl_2_·2H_2_O, 0.2; FeCl_3_, 0.2, and initial pH 4.0 was prepared. The fermentation media was inoculated with 7.5 mL of inoculum and kept in an incubator at 180 rpm and 32 °C 48–120 h.

#### Initial screening of process parameters using Plackett- Burman design (PBD)

A statistical experimental model was created to screen the influential parameters among 10 parameters, for EPS production using PBD with JMP software (Trial Version) as shown in Table S1. The experimental design matrix of PBD with real and coded levels is shown in Table 1. The study was carried out in a 500 mL flask with 100 mL of fermentation media.

**Table 1:** The orthogonal design matrix with real and coded levels of PBD with experimental and predicted EPS yield.

*R*	X_1_	X_2_	X_3_	X_4_	X_5_	X_6_	X_7_	X_8_	X_9_	X_10_	EPS yield (g/L)	
											Experimental	Predicted
1	30 (+)	10 (+)	2 (+)	0.5 (+)	0.5 (+)	0.3 (+)	0.3 (+)	10 (+)	180 (+)	120 (+)	5.94	5.92
2	10 (−)	10 (+)	0.2 (−)	0.5 (+)	0.5 (+)	0.3 (+)	0.1 (−)	5 (−)	0 (−)	120 (+)	6.24	5.74
3	10 (−)	2 (−)	2 (+)	0.1 (−)	0.5 (+)	0.3 (+)	0.3 (+)	5 (−)	0 (−)	48 (−)	7.32	7.31
4	30 (+)	2 (−)	0.2 (−)	0.5 (+)	0.1 (−)	0.3 (+)	0.3 (+)	10 (+)	0 (−)	48 (−)	4.3	3.81
5	10 (−)	10 (+)	0.2 (−)	0.1 (−)	0.5 (+)	0.1 (−)	0.3 (+)	10 (+)	180 (+)	48 (−)	5.94	5.44
6	10 (−)	2 (−)	2 (+)	0.1 (−)	0.1 (−)	0.3 (+)	0.1 (−)	10 (+)	180 (+)	120 (+)	2.96	2.46
7	10 (−)	2 (−)	0.2 (−)	0.5 (+)	0.1 (−)	0.1 (−)	0.3 (+)	5 (−)	180 (+)	120 (+)	4.76	4.75
8	30 (+)	2 (−)	0.2 (−)	0.1 (−)	0.5 (+)	0.1 (−)	0.1 (−)	10 (+)	0 (−)	120 (+)	4.48	4.47
9	30 (+)	10 (+)	0.2 (−)	0.1 (−)	0.1 (−)	0.3 (+)	0.1 (−)	5 (−)	180 (+)	48 (−)	3.42	3.41
10	30 (+)	10 (+)	2 (+)	0.1 (−)	0.1 (−)	0.1 (−)	0.3 (+)	5 (−)	0 (−)	120 (+)	2.08	1.58
11	10 (−)	10 (+)	2 (+)	0.5 (+)	0.1 (−)	0.1 (−)	0.1 (−)	10 (+)	0 (−)	48 (−)	0	0
12	30 (+)	2 (−)	2 (+)	0.5 (+)	0.5 (+)	0.1 (−)	0.1 (−)	5 (−)	180 (+)	48 (−)	7.33	6.83
13	20 (0)	6 (0)	1.1 (0)	0.3 (0)	0.3 (0)	0.2 (0)	0.2 (0)	7.5 (0)	90 (0)	84 (0)	1.22	4.31

X_1_ = Glucose concentration (% w/v); X_2_ = NH_4_Cl (% w/v); X_3_ = K_2_HPO_4_ (% w/v); X_4_ = MgSO_4_·7H_2_O (% w/v); X_5_ = MnSO_4_·H_2_O (% w/v); X_6_ = CaCl_2_ 2H_2_O (% w/v); X_7_ = FeCl_3_ (% w/v); X_8_ = Inoculum size (% v/v); X_9_ = Agitation speed (rpm); and X_10_ = Fermentation time (d)

#### Optimization of the fermentation media using central composite design

Five significant parameters were screened from the Plackett-Burman design (Glucose, NH_4_Cl, K_2_HPO_4,_ MgSO_4_·7H_2_O, MnSO_4_ 2H_2_O) and they are further optimized by central composite design (CCD) to find the optimal conditions. The design matrix was generated using JMP software to elucidate the interaction effect of the above five significant parameters for optimal EPS production. An experiment design of 32 different trials with five different levels was formulated as depicted in Tables S2 and 2.

**Table 2:** The experimental and predicted values of central composite design matrix EPS production.

*R*	X_1_	X_2_	X_3_	X_4_	X_5_	EPS Yield (g/L)	
						Experimental	Predicted
1	40 (+ 1)	2 (+ 1)	1.2 (− 1)	0.8 (+ 1)	0.8 (+ 1)	15	14.92
2	20 (− 1)	2 (+ 1)	1.6 (+ 1)	0.8 (+ 1)	0.8 (+ 1)	13.48	14.25
3	40 (+ 1)	1 (− 1)	1.6 (+ 1)	0.8 (+ 1)	0.8 (+ 1)	15.2	15.34
4	10 (− 2)	1.5 (0)	1.4 (0)	0.6 (0)	0.6 (0)	5.12	5.93
5	50 (+ 2)	1.5 (0)	1.4 (0)	0.6 (0)	0.6 (0)	9.4	9.19
6	20 (− 1)	1 (− 1)	1.6 (+ 1)	0.4 (− 1)	0.8 (+ 1)	12.44	12.93
7	20 (− 1)	2 (+ 1)	1.6 (+ 1)	0.4 (− 1)	0.4 (− 1)	7.2	6.48
8	40 (+ 1)	2 (+ 1)	1.6 (+ 1)	0.4 (− 1)	0.8 (+ 1)	16.1	15.10
9	20 (− 1)	2 (+ 1)	1.2 (− 1)	0.4 (− 1)	0.8 (+ 1)	11.6	10.73
10	30 (0)	1.5 (0)	1.4 (0)	0.6 (0)	0.6 (0)	7.22	8.68
11	20 (− 1)	1 (− 1)	1.6 (+ 1)	0.8 (+ 1)	0.4 (− 1)	10	8.49
12	30 (0)	1.5 (0)	1.4 (0)	0.6 (0)	0.6 (0)	10.38	8.69
13	20 (− 1)	1 (− 1)	1.2 (− 1)	0.8 (+ 1)	0.8 (+ 1)	14	12.74
14	40 (+ 1)	2 (+ 1)	1.6 (+ 1)	0.8 (+ 1)	0.4 (− 1)	10	10.66
15	20 (− 1)	1 (− 1)	1.2 (− 1)	0.4 (− 1)	0.4 (− 1)	3.9	4.97
16	30 (0)	1.5 (0)	1.4 (0)	0.6 (0)	0.2 (− 2)	4.8	4.70
17	30 (0)	1.5 (0)	1.4 (0)	0.2 (− 2)	0.6 (0)	8.16	8.46
18	30 (0)	1.5 (0)	1 (− 2)	0.6 (0)	0.6 (0)	9.2	9.29
19	30 (0)	1.5 (0)	1.4 (0)	0.6 (0)	0.6 (0)	7.23	8.69
20	40 (+ 1)	2 (+ 1)	1.2 (− 1)	0.4 (− 1)	0.4 (− 1)	6.72	7.148
21	30 (0)	1.5 (0)	1.4 (0)	0.6 (0)	0.6 (0)	8	8.69
22	30 (0)	1.5	1.8 (+ 2)	0.6 (0)	0.6 (0)	12.5	13.00
23	40 (+ 1)	1 (− 1)	1.6 (+ 1)	0.4 (− 1)	0.4 (− 1)	8	7.57
24	40 (+ 1)	1 (− 1)	1.2 (− 1)	0.8 (+ 1)	0.4 (− 1)	7.1	7.38
25	30 (0)	2.5 (+ 2)	1.4 (0)	0.6 (0)	0.6 (0)	11.1	11.30
26	40 (+ 1)	1 (− 1)	1.2 (− 1)	0.4 (− 1)	0.8 (+ 1)	12	11.82
27	30 (0)	1.5 (0)	1.4 (0)	0.6 (0)	0.6 (0)	10	8.69
28	30 (0)	1.5 (0)	1.4 (0)	0.6 (0)	1 (+ 2)	16.2	16.90
29	30 (0)	1.5 (0)	1.4 (0)	0.6 (0)	0.6 (0)	9.9	8.69
30	20 (− 1)	2 (+ 1)	1.2 (− 1)	0.8 (+ 1)	0.4 (− 1)	6.5	6.30
31	30 (0)	1.5 (0)	1.4 (0)	1 (+ 2)	0.6 (0)	11.5	11.80
32	30 (0)	0.5(− 2)	1.4 (0)	0.6 (0)	0.6 (0)	9.82	10.22

X_1_ = Glucose concentration (% w/v); X_2_ = NH_4_Cl (% w/v); X_3_ = K_2_HPO_4_ (% w/v); X_4_ = MgSO_4_·7H_2_O (%w/v); and X_5_ = MnSO_4_·H_2_O (% w/v)

### Extraction and purification of EPS

A 50 mL fermentation broth was centrifuged at 10,000 rpm, and 4 °C for 15 min. Next, the chilled absolute ethanol was added to the supernatant in the ratio of 2:1 for the precipitation of the exopolysaccharides. Then, the mixture was kept overnight at 4 °C and later again it was centrifuged at 10,000 rpm at 4 °C for 15 min to collect the EPS in the form of pellets. The dry weights of the pellets were noted to estimate the amount of crude EPS yield. The EPS extracted was further purified by dissolving in distilled water and centrifuged at 10,000 rpm at 4 ℃ for 15 min^20,21,24,26^. Finally, the purified EPS was lyophilized for further characterization and application studies.

### Characterization of the EPS produced by *Sporosarcina psychrophila* MTCC − 2908

The purified EPS was characterized using different thermoanalytical techniques, including SEM, AFM, XRD, TGA, and FTIR, offer insights into the structural, morphological, and thermal stability of EPS. Collectively, these techniques provide compressive chemical characterization, including identification of monomeric residues, glycosidic linkages, and branching pattern^25,26^.

#### Fourier transform infrared spectroscopy (FTIR)

FTIR spectroscopy is used to detect functional groups and molecular bonds based on characteristic absorption frequencies. This was acquired in transmittance mode with a Shimadzu spectrophotometer The EPS sample was transferred into the container and 50 scans were performed in 4 cm^− 1^ resolution.

#### ThermoGravimetric analysis (TGA)

TGA was used to measure the mass over time as its temperature changes. TGA of the exopolysaccharide was performed using Hitachi STA7200 Thermal Analysis System. The EPS sample of 1.0 mg was heated in a linear range rate of 20 °C over a temperature range of 40–730 °C under nitrogen flow of 200 mL, and the corresponding weight loss was monitored and recorded.

#### Atomic force microscopy (AFMS)

AFMS is a technique used for the imaging of the surface of the samples. The atomic force microscopy of the EPS was carried out as described elsewhere^17^. Briefly, the glass slides were treated with a blend of 15 mL HCl and 5mL HNO_3_ for 30 min. Meanwhile, the EPS sample was subjected to a mixture of 20 mL H_2_SO_4_ and 5 mL H_2_O_2_ for 30 min. Next, the glass slides were rinsed with double distilled water, and stored for further use. Then, a fresh EPS solution was prepared by dissolution in double distilled water. Approximately 10 µL of EPS sample was deposited onto the surface of glass slide and allowed to air dry at room temperature. Finally, the AFMS images were captured using the Innova SPM Atomic Force Microscope.

#### X-ray diffraction (XRD)

To determine the physical characteristics of the purified EPS, XRD was recorded using a powder diffractometer. Scanning was carried out at a range of 2$\:\theta\:$ angles (2–70 °C). The crystallinity index was found with the ratio between the area under the crystalline peaks and the total area of the amorphous and crystalline peaks as shown below:^27^

$$CI\, \% = \frac{{\sum {Area\,Under\,Crystalline\,peaks} }}{{\sum {Area\,Under\,Crystalline\,peaks} \, + \, \sum {Area\,under\,Amorphous\,peaks} }} \times 100$$

#### Scanning electron microscopy–energy-dispersive X-ray analysis

Scanning electron microscopy of lyophilized EPS samples was carried out using Zeiss EVO 18 Special edition Scanning Electron Microscope at 15 KeV accelerating voltage and images at magnifications of 3000×, 5000× and 10,000× were recorded. For SEM analysis of EPS was made conductive by gold-coating, using quorum gold sputtering unit. The elemental mapping of the EPS was performed by energy-dispersive X-ray spectroscopy for analysis of carbon/oxygen/nitrogen/phosphorus and sulphur composition. The X-ray spectrum of the elements was obtained at an accelerating voltage of 15 KeV^28^.

#### Total antioxidant capacity (TAC) of EPS

The TAC test measures how a sample removes free radicals, and thereby assess the antioxidant capacity of biological samples. TAC was prepared by dissolving 4 mM Ammonium molybdate, 28 mM Sodium sulphate, and 45 mL of sulfuric acid in 250 mL of water. Different amounts of EPS ranging from (300–1200 µg) was dissolved in 1mL of the TAC mix and kept for incubation at room temperature for 1 h and then absorbance was measured at 695 nm using a spectrophotometer. Ascorbic acid was used as standard (10–40 µg)^16^.

#### Reducing power of EPS

The Reducing power of the EPS was assessed using a ferric reducing antioxidant power (FRAP) assay. This assay reflects the electron-donating ability of the EPS, which is crucial for neutralizing free radicals and preventing oxidative damage in biological and food systems^21,29^. Different concentrations of EPS (300–500 µg) dissolved in 1 mL of distilled water were combined with 2.5 mL of 1% potassium ferricyanide. This mixture was then subjected to incubation at 50 °C for 20 min. Subsequently, 2.5 mL of 10% trichloroacetic acid was introduced and the resulting mixture was centrifuged at 3000 rpm for 10 min. To the 2.5 mL supernatant, 2.5 mL of distilled water and 0.5 mL of ferric chloride (0.1%) were added. Then, incubated for 10 min, and later the absorbance was measured at 700 nm using a spectrophotometer with ascorbic acid as standard. A higher absorbance value indicates greater reducing power^29,30^.

## Result and discussion

### Initial screening of fermentation media using Plackett-Burman design

In the experimental PBD, the EPS yield was ranged from 0 to 7.33 g/L, which indicates the significance of parameters selected for the optimization of production of EPS (Table 1). This widespread distinction too imitates the importance of fermentation media optimization for accomplishing higher yield. The PBD showed 6-fold increase in EPS yield when compared to unoptimized condition (Table 1; Run order 12). This result is in good agreement with the results reported elsewhere^31–33^. The first order mathematical model of PBD based on the regression analysis with coded values is depicted below:

1 $$\begin{aligned} Y & = 2.619 + 0.017\,{\text{ X}}_{1} + 0.013\,{\text{ X}}_{2} + 0.227\,{\text{ X}}_{3} + 1.524\,{\text{ X}}_{4} + 8.757\,{\text{ X}}_{5} + 6.01\,{\text{ X}}_{6} \\ & \quad + 6.356\,{\text{ X}}_{7} {-}0.165\,{\text{X}}_{8} + 0.006\,{\text{X}}_{9} + 0.0003\,{\text{X}}_{{10}} \\ \end{aligned}$$

where Y represents EPS yield (g/L); X_1_, Glucose; X_2_, NH_4_Cl; X_3_, K_2_HPO_4_; X4, MgSO_4_·7H_2_O; X_5_, MnSO_4_·H_2_O; X_6_, CaCl_2_·2H_2_O; X_7_, FeCl_3_; X_8_, Inoculum size; X_9_, Agitation Speed; and X_10_, Fermentation time.

**Fig. 2:**
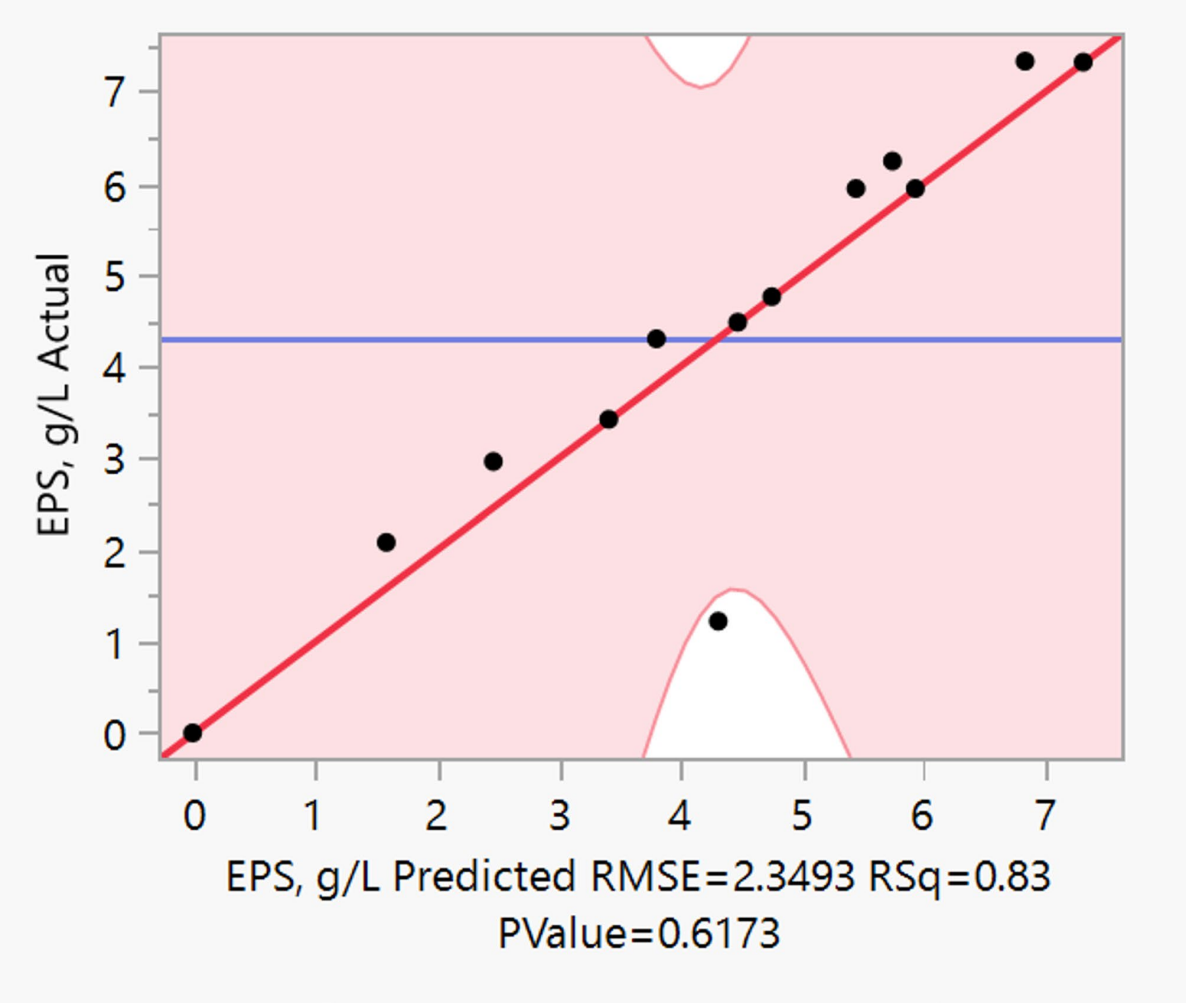
A normal plot between actual and predicted values of EPS yield of PBD.

**Fig. 3:**
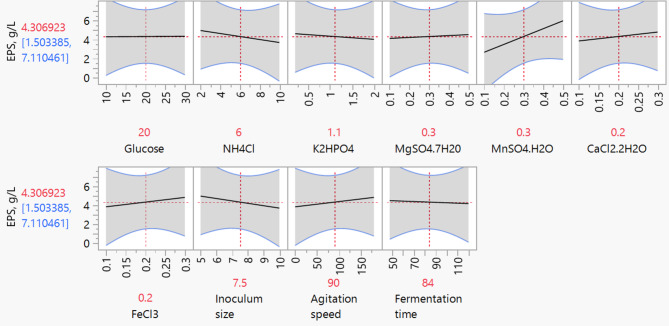
A prediction profiler generated by PBD for the EPS production.

The model (Eq. 1) exhibited good predictability with a coefficient of determination (R^2^) of 0.83, indicating that 83% of the variation in EPS yield could be explained by the linear combination of the variables. The minimal residuals observed between experimental and Predicted values further validated model adequacy (Fig. 2). The prediction profiler generated by JMP software screened five significant parameters such as Glucose, NH_4_Cl, K_2_HPO_4_, MgSO_4_·7H_2_O, and MnSO_4_·H_2_O (Fig. 3). Further, these significant parameters were optimized with central composite design to find the optimal values for an enhanced EPS yields.

### Central composite design (CCD)

The significant parameters such as glucose concentration, NH_4_Cl, K_2_ HPO_4_, MgSO_4_·7H_2_O, and MnSO_4_·H_2_O, were further optimized for the production of EPS using CCD at 5 levels and 6 central points with 33 different experimental runs by *Sporosarcina psychrophila* MTCC–2908. The CCD matrix with coded and uncoded values of above-mentioned parameters with EPS yield are shown in Tables S2 and 2. These data showed that the EPS yield increased to 16.2 g/L (Run 28) to 3.9 g/L (Run 5) through submerged fermentation. The results of the experimental design matrix were fitted with a polynomial equation as a function of five parameters with coded values and are shown in Eq. 2 for EPS production.

2 $$\begin{aligned} Y & = - 12.696 + 0.081\,{\text{ X}}_{1} + 0.543\,{\text{ X}}_{2} + 4.625\,{\text{X}}_{3} + 4.166\,{\text{X}}_{4} + 15.25\,{\text{X}}_{5} - 0.002\,\left( {{\text{X}}_{1} } \right)^{2} + 0.088\,{\text{X}}_{1} \,{\text{X}}_{2} + 2.071\,\left( {{\text{X}}_{2} } \right)^{2} + 0.042\,{\text{X}}_{1} \,{\text{X}}_{3} \\ & \quad - 1.05\,{\text{X}}_{2} \,{\text{X}}_{3} + 15.383\,\left( {{\text{X}}_{3} } \right)^{2} - 0.136\,{\text{X}}_{1} \,{\text{X}}_{4} - 4.125\,{\text{ X}}_{2} \,{\text{X}}_{4} - 5.375\,{\text{X}}_{3} \,{\text{X}}_{4} + 9.008\,\left( {{\text{X}}_{4} } \right)^{2} + 0.08\,{\text{ X}}_{1} \,{\text{X}}_{5} + 0.7\,{\text{X}}_{2} \,{\text{X}}_{5} \\ & \quad - 9.937\,{\text{X}}_{3} \,{\text{X}}_{5} - 3.5\,{\text{X}}_{4} \,{\text{X}}_{5} + 13.196\,\left( {{\text{X}}_{5} } \right)^{2} \\ \end{aligned}$$

where Y, EPS yield of *Sporosarcina psychrophila* MTCC–2908 (g/L); X_1_, Glucose concentration (% w/v); X_2_, NH_4_Cl (% w/v); X_3_, K_2_HPO_4_ (% w/v); X_4_, MgSO_4_·7H_2_O (% w/v); X_5_, MnSO_4_·H_2_O (% w/v).

The results of the statistical analysis of variance (ANOVA) obtained in this study for the production of EPS are shown in Table 3. The results are in good agreement with the general facts of higher F value (F = 26.052, *p* < 0.0001), and R^2^ (0.96), which specifies a better fit and suggest strong explanatory power with low prediction error (RMSE = 1.106). *P*-values < 0.05 indicate that the model terms are significant. In this study, all linear and quadratic terms were significant except X_2_, and X_1_^2^, X_4_^2^, respectively (*P* < 0.05). Whereas all the parameters did show any interaction effect for EPS production. The lack of fit was insignificant (*P* = 0.9516) indicating that the model adequately captures the variability in the data without systematic error (Table 3). A 95% confidence interval was used throughout the model fitting, ensuring that parameter estimates were statistically robust. The confidence intervals and low *P*-value (< 0.05) associated with most model terms reinforce the reliability of the optimized parameters.

**Table 3:** Analysis of variance values for the quadratic regression model obtained from CCD employed in the optimization of fermentation medium for the production of EPS from *Sporosarcina psychrophila* MTCC–2908.

Source	DF	Sum of squares	Contribution %	F Ratio	Prob > F
Model	11	311.573	93.48	26.052	< 0.0001*
X_1_	1	15.941	4.78	14.662	0.0010*
X_2_	1	1.771	0.531	1.6291	0.2164
X_3_	1	20.535	6.161	18.8870	0.0003*
X_4_	1	16.667	5.00	15.3291	0.0009*
X_5_	1	223.260	66.981	205.3423	< 0.0001*
X_1_^2^	1	2.335	0.701	2.1479	0.1583
X_1_ × _2_	1	3.133	0.94	2.8815	0.1051
X_2_^2^	1	7.866	2.36	7.2347	0.0141*
X_3_^2^	1	11.107	3.332	10.2155	0.0045*
X_4_^2^	1	3.809	1.143	3.5031	0.0760
X_5_^2^	1	8.173	2.45	7.5168	0.0126*
Lack of fit	15	10.998	3.30	0.341	0.9516
Pure error	5	10.747	3.224		
C. Total	31	333.319	100		
Total error	20	21.745			

X_1_ = Glucose concentration (% w/v); X_2_ = NH_4_Cl (% w/v); X_3_ = K_2_HPO_4_ (% w/v); X_4_ = MgSO_4_·7H_2_O (% w/v); and X_5_ = MnSO_4_·H_2_O (% w/v).

R^2^ = 0.96; R^2^-Adj = 0.89; and RMSE = 1.106

*Statistically significant (95% confidence interval).

The optimization study revealed that macro- and micronutrients significantly influence production by *Sporocarcina psychrophile* MTCC-2908. Glucose acts as a primary carbon source, promoting biosynthesis via overflow metabolism under nutrient-limited conditions^34,35^. NH_4_Cl, a nitrogen source, is essential for protein synthesis, but excess nitrogen may suppress EPS production due to biomass favouring over metabolite synthesis^2^. K_2_HPO_4_ serves as a phosphorus source and buffering agent, maintaining pH and supporting nucleic acid synthesis. MgSO_4_·7H_2_O enhances enzyme activity and stabilizes ribosomes, while MnSO_4_·H_2_O, a redox cofactor, likely induces oxidative stress, triggering stress-mediated EPS biosynthesis. The elevated EPS yield under optimal conditions suggests that certain concentrations of these nutrients impose mild stress, shifting metabolism toward exopolysaccharide production^34^. These findings align with previous CCD-optimized studies on EPS production in *Leuconostoc citreum*^36^ and *Virgibacillus dokdonensis*^37^.

**Fig. 4:**
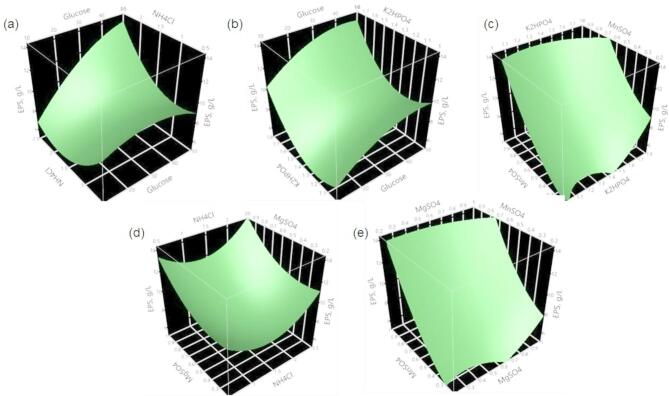
Surface plots showing the interaction effects of parameters on EPS yield produced by *Sporosarcina psychrophila* MTCC–2908 under submerged fermentation with the remaining parameters held constant at the centre level of the CCD. (**a**) Glucose and NH_4_Cl, (**b**) Glucose and K_2_HPO_4_, (**c**) K_2_HPO_4_ and MnSO_4_, (**d**) NH_4_Cl and MgSO_4_·7H_2_O, and (**e**) MgSO_4_·7H_2_O and MnSO_4_·H_2_O.

**Fig. 5:**
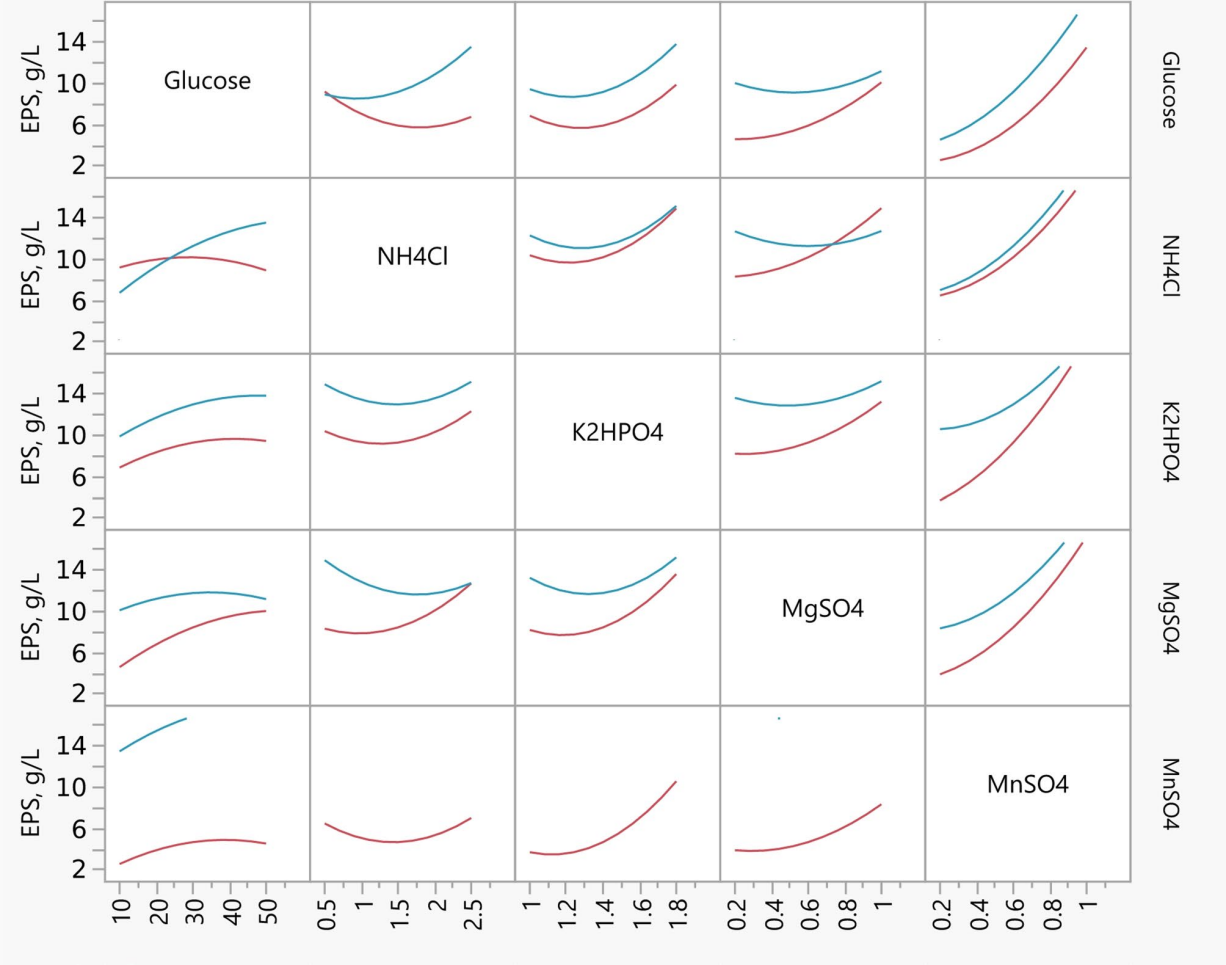
Interaction Profiles of all the parameters for an optimized EPS yield of *Sporosarcina psychrophila* MTCC–2908 with CCD.

In order to visualize the interaction effects between each parameter on EPS production by *Sporosarcina psychrophila* MTCC–2908, three dimensional surface plots are shown graphically in Fig. 4. The surface plot of the parameters Glucose and NH_4_Cl, Glucose and K_2_HPO_4_, K_2_HPO_4_ and MnSO_4_, and MgSO_4_ and MnSO_4_ showed less interaction with an increase in EPS yield at their hold values. While the interaction between the remaining parameters was not statistically significant, but the individual parameters except NH_4_Cl are statistically significant (Table 3). These results are in good agreement with the interaction plot shown in Fig. 5, in which the good interaction between Glucose and NH_4_Cl, NH_4_Cl and MgSO_4_, was observed; whereas a very weak interaction was observed between Glucose and MgSO_4_·7H_2_O; K_2_HPO_4_ and NH_4_Cl, NH_4_Cl and MgSO_4_. The combination of their lower and higher levels may not have shown any interactions, but their lower levels may be prominent for EPS production. The proposed second order regression model (Eq. 2) was solved using the prediction profiler tool in JMP software for maximum EPS yield and optimum levels of each parameter in uncoded units were as follows (% w/v): Glucose concentration = 50, NH_4_Cl = 2.5, K_2_HPO_4_ = 1.8, MgSO_4_·7H_2_O = 1.0, and MnSO_4_·H_2_O = 1.0; all of them were found within the experimental range. Under these optimum conditions, the predicted EPS yield was 24.29 g/L.

In this study, CCD has found to be a valuable tool for optimizing the fermentation media composition in production of EPS. Andrew et al.^37^, enhanced the EPS yield of *Virgibacillus dokdonensis* VITP14 using RSM-CCD technique; and showed the maximum yield of 26.5 g/L at optimal conditions of glucose 20 g/L, peptone 10 g/L, and NaCl 50 g/L. whereas Ge et al.^38^, optimized the production of exopolysacchrides with *Leuconostoc citreum* BH10 and CCD, which showed the maximum EPS yield was up to 55.96 g/L. Similarly, Ahmed et al.^39^, reported the production of EPS using *L. kefiranofaciens* ZW3, and optimized its production with CCD and showed the optimal level of 1.602 g/L at lactose (0.36%), tryptophan (0.23%), and pH (5.17).

**Fig. 6:**
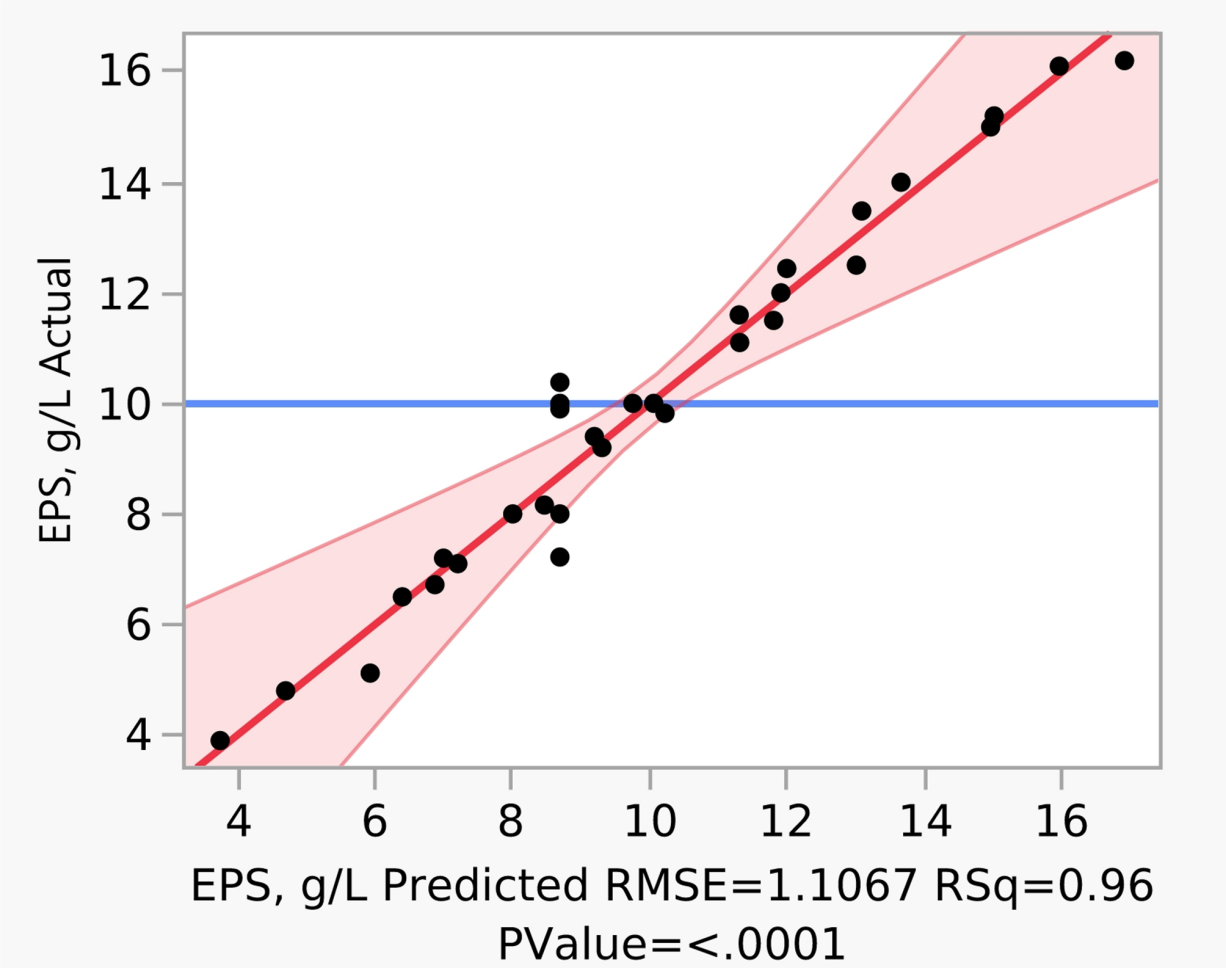
A normal plot between experimental and predicted EPS yield of *Sporosarcina psychrophila* MTCC–2908 with the central composite design.

In the current study, the maximum EPS yield of *Sporosarcina psychrophila* MTCC–2908 was found to be 21.62 g/L. This yield was comparatively higher than that reported for different bacterial strains^39–41^. In order to validate the mathematical model Eq. (2), experiments were carried out at the above-mentioned optimized values in triplicates. The experimental value of EPS yield from *Sporosarcina psychrophila* MTCC–2908 was found to be 21.62 g/L under optimized conditions. The good correspondence between experimental and predicted values confirms the acceptability of the model (Fig. 6). This optimization approach led to the enhancement of EPS yield by *Sporosarcina psychrophila* MTCC–2908 from 1.22 g/L (unoptimized medium) to 21.62 g/L (optimized medium), a 17.72-fold increase. The result of this study suggests that a statistical optimization is a useful approach for determining the optimum conditions in submerged fermentation system. No literature is available on *Sporosarcina psychrophila* MTCC–2908 for the production of EPS.

### Characterization of the EPS

#### Fourier transform infrared spectroscopy (FTIR)

**Fig. 7:**
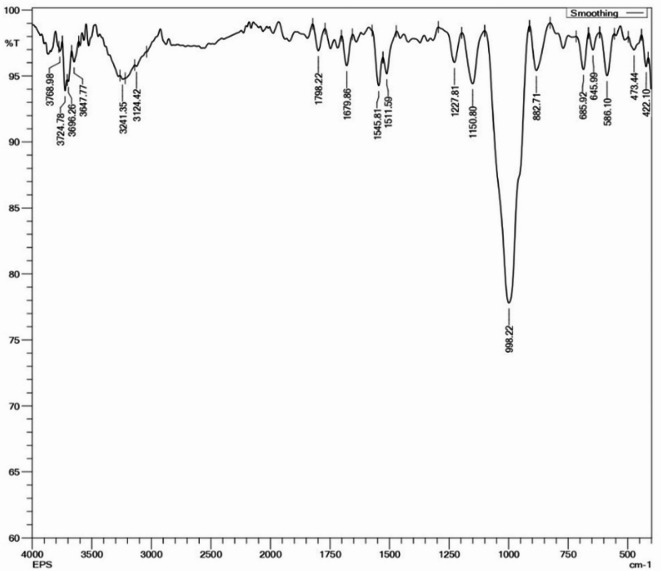
The FTIR Spectrum of the purified EPS of *Sporocarcina psychrophile* MTCC–2908.

FTIR is an effective technique to detect functional group bonds vibrate at the characteristic frequencies. It can be employed to detect functional groups and to characterize covalent bonds. The Fig. 7 shows the FTIR spectrum of the purified and lyophilized EPS. The broad stretching in the range between 3000 and 3500 cm^− 1^ represents the vibrations of hydroxyl groups. This is a defining feature of polysaccharides, reflecting the presence of abundant hydroxyl groups involved in intramolecular and intermolecular hydrogen bonding. The shoulder-like absorption in this region also suggests possible interactions with water molecules, either trapped or structurally bound^42^. The band between 2800 and 2900 cm^− 1^ is ascribed to C–H stretching and vibrations of aliphatic –CH and –CH_2_ groups, commonly found in sugar ring structure^43^. A notable absorption at ~ 1790 cm^− 1^ is indicative of carbonyl (C=O) stretching, possibly from uronic acids or esterified carboxylic groups, implying that the EPS may contain acidic sugars such as glucuronic or galacturonic acid. This is supported by another shoulder observed at ~ 1670 –1645 cm^− 1^, which can be assigned to C=O stretching or amide I vibrations if minor proteinaceous residues are present^44^. The band at 1150 cm^− 1^ characterizes the presence of dextran α (1◊ 6) exopolysaccharide^45^. The absorption band at 998 cm^− 1^ indicates the vibrations of glycosidic linkage C–O– C^46^. The characteristic band at absorption 882 cm^− 1^ indicates the presence of a furanoid ring of sugar units^43^. The results of FTIR spectrum confirms the presence of exopolysaccharide.

#### Thermogravimetric analysis (TGA)

**Fig. 8:**
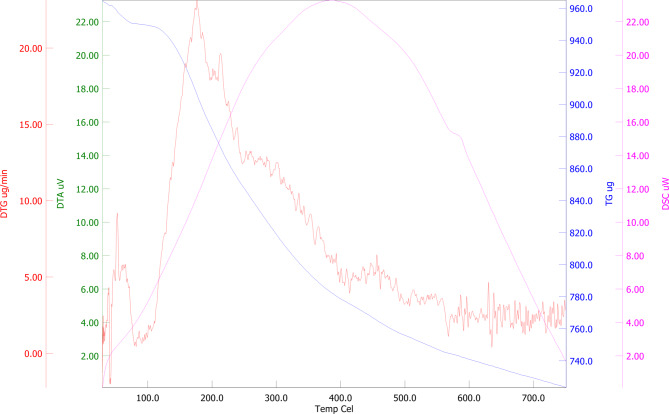
The graphical representation of TGA of produced EPS.

The graphical representation of TGA of produced EPS is depicted in Fig. 8. The initial weight loss of approximately 5–10% was observed in the temperature range of 40 to 120 °C, corresponding to the evaporation of physically adsorbed water and moisture bound through hydrogen bonding. This is typical of polysaccharides due to their hydrophilic nature and high hydroxyl group content^47,48^. The second phase of the degradation step started from 150 to 350 °C. This is the most significant phase of degradation, accounting for the primary weight loss (~ 40–60%). The DTG curve (red) displays a pronounced peak centred around ~ 230 °C, indicative of the maximum degradation rate. This stage is associated with the decomposition of the EPS backbone, specifically the depolymerisation of glycosidic linkages and cleavage of monosaccharide units. The corresponding endothermic peak in the DTA curve (magenta) aligns with this degradation, further confirming a breakdown of the polymer matrix. Finally, the gradual weight loss of ~ 10–15% was observed in the third stage ranged from 400 to 700 °C, reflecting the carbonization and decomposition of more thermally resistant fractions such as aromatic residues or residual cross-linked structures. The levelling off of the TGA curve beyond 650 °C suggests a thermally stable char residue. The onset of significant degradation above 200 °C indicates good thermal stability, which is advantageous for industrial applications. The presence of a substantial carbonaceous residue after 700 °C implies a robust polysaccharide matrix with potential antioxidant. Fagerson^47^ reported that the EPS was exposed to varying temperatures, it undergoes distinct processes. Initially, as the temperature rises, gelatinization and swelling are the primary occurrences. Subsequent temperature elevation leads to dehydration and the pyrolysis of the exopolysaccharide.

#### Atomic force microscopy (AFM)

**Fig. 9:**
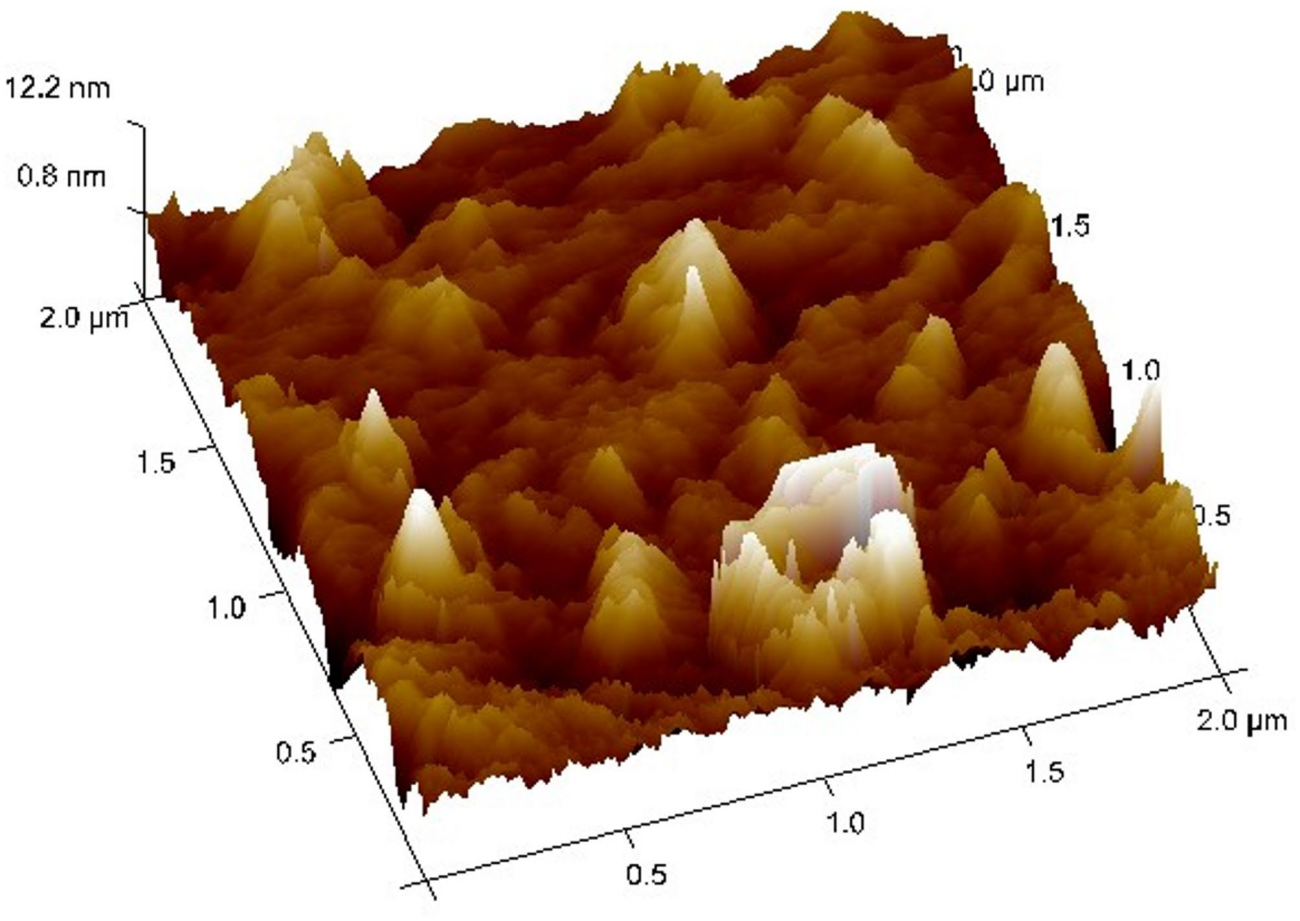
The 3 D topographical representation of extracted EPS with AFM.

The AFM has been used to analyze the surface topography of the extracted exopolysaccharides (EPS) in several studies^48,49^. The topographical image of the produced EPS under AFM is shown in Fig. 9. The EPS of *Sporocarcina psychrophile* MTCC–2908 revealed a non-uniform and heterogeneous surface with well-defined nanoscale peaks and valleys. The average surface roughness was calculated to be approximately 12.2 nm, indicative of significant irregularities. This observation is in good agreement with Ahmed et al.^49^. The observed nanoscale roughness suggests a highly textured EPS surface, which is likely to enhance its functional properties. Increased surface area, due to roughness, can provide more binding sites for interactions with external agents such as heavy metals, dyes, or nutrients. This topographical feature may also facilitate microbial attachment and biofilm formation, further emphasizing the ecological and industrial relevance of the extracted EPS.

#### Scanning electron microscope and energy-dispersive X-ray analysis

**Fig. 10:**
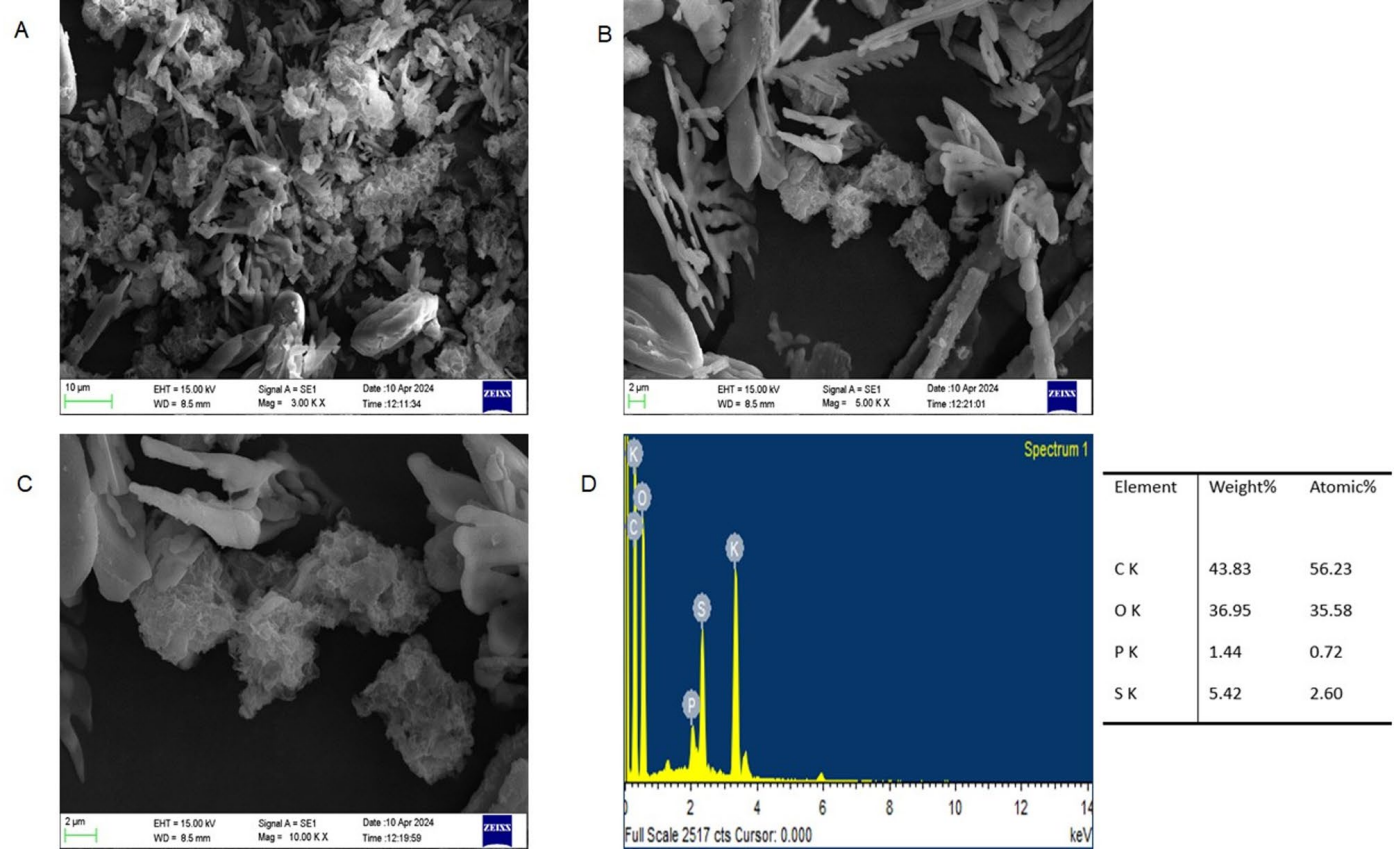
SEM images of produced EPS under different magnifications of 3000X (**A**), 5000X (**B**), and 10000X (**C**); and EDS spectrum with different elements (**D**).

The Scanning Electron Micrographs in different magnifications of 3000×, 5000× and 10,000× represented in Fig. 10. At lower magnifications (3000x), the EPS displayed irregular, flake-like aggregates typical of polysaccharide-dominated biopolymers. Higher magnifications (5000× and 10000×) revealed more intricate structures, including needle-like crystalline features interspersed with amorphous clusters. The presence of both crystalline and amorphous regions suggests a heterogeneous polymeric matrix comprising multiple EPS constituents, such as carbohydrates, proteins, and possibly nucleic acids. The amorphous zones could be associated with loosely bound or highly hydrated polymer fractions, while the crystalline domains may represent ordered regions formed by specific polysaccharides or by the interaction of EPS with inorganic components. The EPS had a porous web-like structure like the bacteria *Lactobacillus plantaram*^48,50^.

The EDX was performed to analyze the elemental composition of EPS. The EPS’s organic composition was supported by the spectrum’s significant abundance of carbon (43.83%) and oxygen (369.5%). Interestingly, elements like sulfur (5.42%) and phosphorus (1.44%) were also found. The substantial amounts of carbon and oxygen are consistent with the biochemical makeup of EPS, which is mainly composed of proteins and carbohydrates. While sulfur may be found as a component of sulfated polysaccharides or amino acids like cysteine and methionine, the detection of phosphorus indicates the potential integration of phosphorylated substances, such as nucleotides or phospholipids. Because they can support metal ion chelation, antibacterial activity, and electrostatic interactions with charged molecules, these heteroatoms are functionally significant.

Finally, the AFM and SEM-EDX analyses confirmed that the extracted EPS possesses a complex and functional morphology enriched with chemically significant elements. These characteristics make it a promising candidate for various industrial and environmental applications, including as a bioflocculant, bioadsorbent, or stabilizer in food and pharmaceutical formulations.

#### X-ray diffraction

**Fig. 11:**
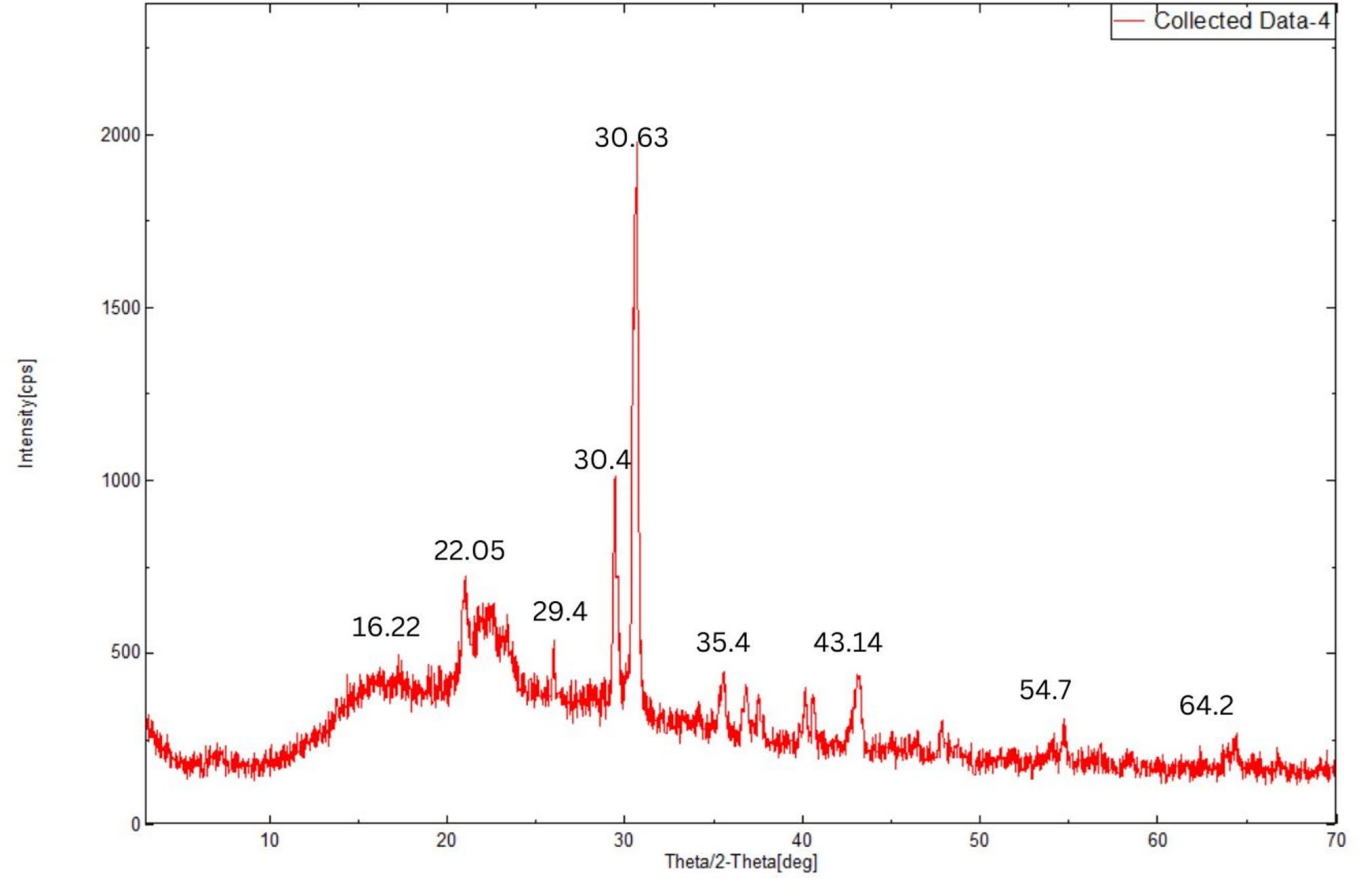
The XRD spectrum of EPS.

The XRD pattern of the EPS from *Sporosarcina psychrophila* MTCC–2908 revealed a semi-crystalline nature, as evidenced by both broad and sharp diffraction peaks as shown in Fig. 11. Notably, the broad peaks at 16.22°, 22.05°, and 29.4° indicate amorphous regions typically associated with randomly coiled polysaccharide chains. A sharp, intense peak at 30.63°, along with minor peaks at 30.4°, 35.4°, 43.14°, 54.7°, and 64.2°, suggests the presence of ordered, crystalline domains within the EPS matrix (Fig. 11). The Crystallinity Index Percent (*CI* %) was found to be 50.41% (*CI* = 0.54), from which we can conclude that the EPS is semi-crystalline in nature^27^. The semi-crystalline structure may contribute to the EPS’s functional properties such as mechanical stability, thermal resistance, and controlled solubility, which are advantageous for food, pharmaceutical, and biomedical applications^51^.

#### Analysis of total antioxidant capacity and reducing power of EPS

**Fig. 12:**
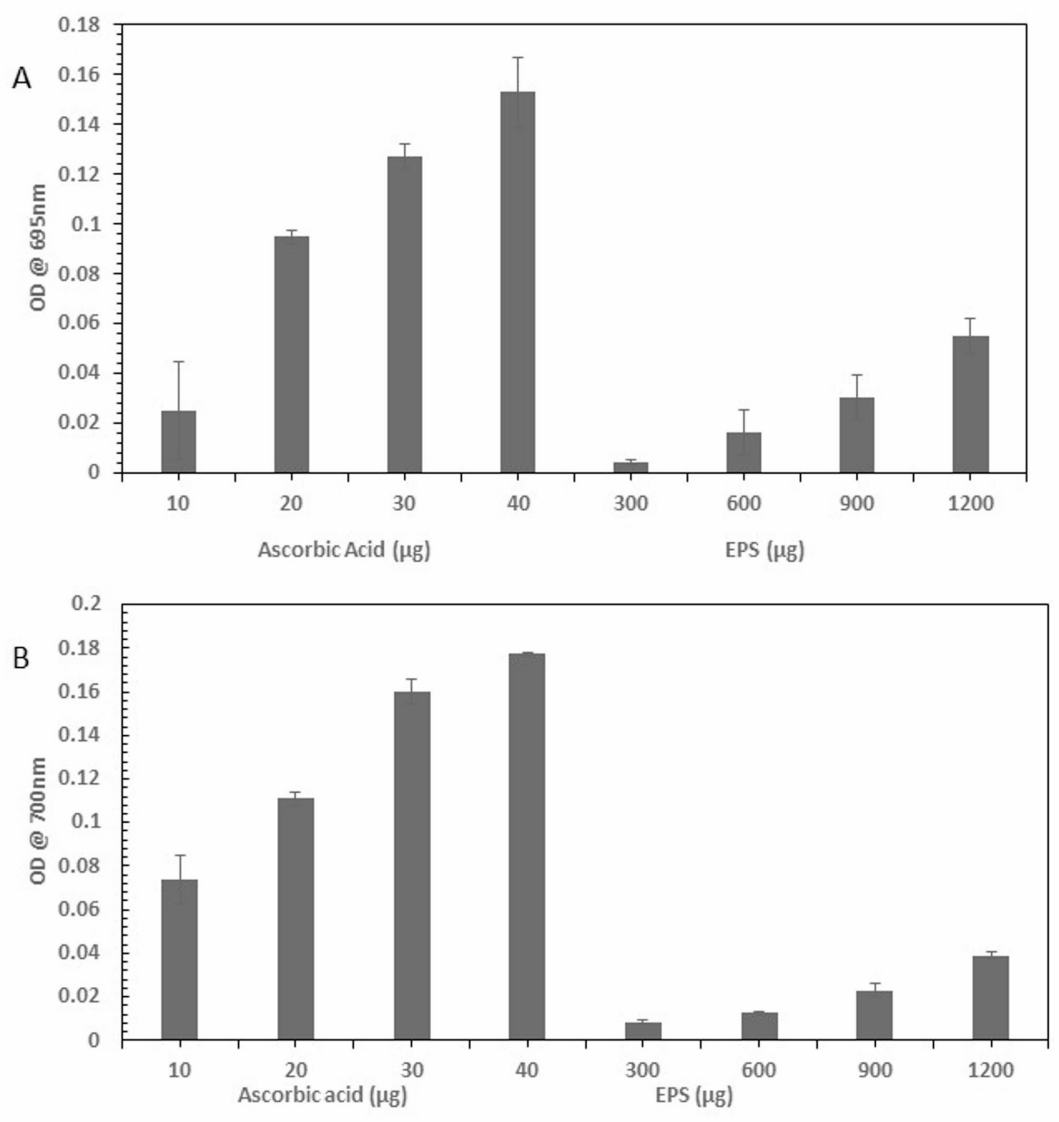
Analysis of total antioxidant capacity (**A**), and reducing power (**B**) of EPS.

The antioxidant capacity of the EPS is illustrated in Fig. 12A. The antioxidant potential of EPS exhibited a dose-dependent activity in the concentration range (300–1500 µg). While its activity was lower to moderate than that of the reference antioxidant, ascorbic acid; the exopolysaccharide (EPS) showed a concentration-dependent increase in both reducing power (OD @ 700 nm) and overall antioxidant capacity (OD @ 695 nm) (Fig. 12A,B). The structural variations in the active moieties are probably the cause of this discrepancy. While EPS, a large molecular weight polymer, depends on distinct, frequently less effective methods, ascorbic acid, a small molecule antioxidant, rapidly distributes electrons and hydrogen atoms.

The presence of hydroxyl groups on the sugar monomers, which can donate electrons to neutralize free radicals or decrease metal ions, may be responsible for the antioxidant and reducing properties of EPS. Furthermore, EPS may work by chelating transition metal ions (such as Fe^2+^) to prevent Fenton-type reactions and the production of reactive oxygen species (ROS) that follow. If present, structural elements like conjugated proteins or phenolics, uronic acids, or sulfate groups may increase the electron-donating capacity. These findings are consistent with those reported previously^17^. The pharmaceutical relevance of EPSs extends beyond antioxidant potential alone. EPS are widely recognized for their biocompatibility, biodegradability, film-forming ability, and immunomodulatory effects, which support their utility as drug delivery matrices, wound-healing agents, or vaccine adjuvants. The low to moderate antioxidant activity observed may still contribute to ancillary benefits in such applications.

The EPS from *Sporocarcina psychrophila* MTCC–2908 exhibited distinctive structural characteristics compared to EPS from other microbial sources. FTIR analysis confirmed hydroxyl, carboxyl, and α-glycosidic linkages, typical of neutral and acidic polysaccharides, but lacked sulfated groups, unlike marine bacterial EPS^26,43^. TGA revealed good thermal stability with a major degradation peak around 300 °C, comparable to EPS from *Bacillus subtilis*^12,16^. Like dextran-type EPS^45^AFM showed a smooth, branched surface morphology, while SEM images revealed a porous, fibrillar network, similar to EPS produced by *Lactobacillus* spp^1^. EDX analysis confirmed carbon and oxygen as major elements, aligning with typical polysaccharide compositions. XRD patterns displayed broad peaks, indicating an amorphous nature, in contrast to the semi-crystalline structure seen in xanthan gum^40^. Together, these characterizations suggest a thermally stable, amorphous EPS with potential applicability in food, pharmaceutical, or biodegradable material industries where non-sulfated, neutral polysaccharides with moderate thermal resistance are advantageous.

## Conclusion

The strain *Sporocarcina psychrophile* MTCC–2908 showed a significant potential for the production of EPS under optimized fermentation conditions. The two-step statistical optimization method i.e. PBD and CCD resulted in a 17.72-fold increase (21.62 g/L) under submerged fermentation at 48 h. The low fermentation period of 48 h may contribute to reduced production time and associated operational costs, which could be advantageous for future scale-up. The purified EPS was characterized using FTIR, TGA, AFM, SEM, and XRD, total antioxidant capacity and the reducing capacity of the EPS. The FTIR spectrum showed the presence of furanoid ring of sugar units; TGA showed peak degradation of EPS after 176.9 °C; AFM revealed that EPS from *Sporocarcina psychrophile* MTCC–2908 was tightly packed with surface roughness and had a strong affinity towards water. The EPS showed a three-dimensional structure with irregular lumps and with a coarse surface and amorphous surface roughness under SEM. The XRD spectrum of the EPS exhibited semi crystalline in nature. Further, the EPS showed the moderate amount of antioxidant and reducing power capacities, and this property can be explored in pharmaceutical sector. Therefore, the EPS extracted from bacteria *Sporocarcina psychrophila* (MTCC–2908) can be a potent candidate for further research on its therapeutical application and exploitation for different industrial uses. Future studies should focus on extracting highly pure EPS with advanced purification techniques ion-exchange chromatography or gel-filtration chromatography, and also advanced structural characterization of the EPS using techniques like NMR and GC-MS to better understand its molecular composition. Antimicrobial and anti-inflammatory assays can be performed to explore its potential in therapeutic applications.

## Electronic supplementary material

Below is the link to the electronic supplementary material.

Supplementary Material 1

## Data Availability

Data can be made available from Subbalaxmi Selvaraj upon reasonable request.
