# Supplementary material for: Enhanced production and structural characterization of exopolysaccharide from Sporocarcina psychrophile MTCC 2908 through two step optimization for therapeutic evaluation
Source: Sci Rep. 2025 Jul 17;15:25899. doi: 10.1038/s41598-025-10392-5 (PMC12267618; doi:10.1038/s41598-025-10392-5)
Supplement: Supplementary file 1 — Supplementary Material 1 [file 41598_2025_10392_MOESM1_ESM.docx]

**Enhanced production and structural characterization of exopolysaccharide from** ***Sporocarcina psychrophile* MTCC 2908 through two step optimization for therapeutic evaluation**

**Supplementary material**

Table S1: Levels of independent parameters used in PBD for the production of EPS

| Parameters | High (+1) | Low (-1) | Units |
| --- | --- | --- | --- |
| Glucose | 30 | 10 | % (w/v) |
| NH_4_Cl | 10 | 2 | % (w/v) |
| K_2_HPO_4_ | 2 | 0.2 | % (w/v) |
| MgSO_4_.7H_2_O | 0.5 | 0.1 | % (w/v) |
| MnSO_4_.H_2_O | 0.5 | 0.1 | % (w/v) |
| CaCl_2_.2H_2_O | 0.3 | 0.1 | % (w/v) |
| FeCl_3_ | 0.3 | 0.1 | % (w/v) |
| Inoculum Size | 10 | 5 | % (w/v) |
| Agitation Speed | 180 | 0 | rpm |
| Fermentation time | 120 | 48 | h |

Table S2: The different levels of parameters chosen in CCD for EPS production

| Parameters | -2 | -1 | 0 | +1 | +2 |
| --- | --- | --- | --- | --- | --- |
| Glucose, % (w/v) | 10 | 20 | 30 | 40 | 50 |
| NH_4_Cl, % (w/v) | 0.5 | 1 | 1.5 | 2 | 2.5 |
| K_2_HPO_4_, % (w/v) | 1 | 1.2 | 1.4 | 1.6 | 1.8 |
| MgSO_4_.7H_2_O, % (w/v) | 0.2 | 0.4 | 0.6 | 0.8 | 1 |
| MnSO_4_.2H_2_O, % (w/v) | 0.2 | 0.4 | 0.6 | 0.8 | 1 |
